# Mechanobiology in Cardiovascular Disease Management: Potential Strategies and Current Needs

**DOI:** 10.3389/fbioe.2016.00079

**Published:** 2016-10-10

**Authors:** Samantha K. Atkins, Andrew McNally, Philippe Sucosky

**Affiliations:** ^1^Department of Aerospace and Mechanical Engineering, University of Notre Dame, Notre Dame, IN, USA; ^2^Department of Materials and Mechanical Engineering, Wright State University, Dayton, OH, USA

**Keywords:** mechanobiology, cardiovascular disease, hemodynamics, wall shear stress, translational research

## Introduction

**Figure 1:**
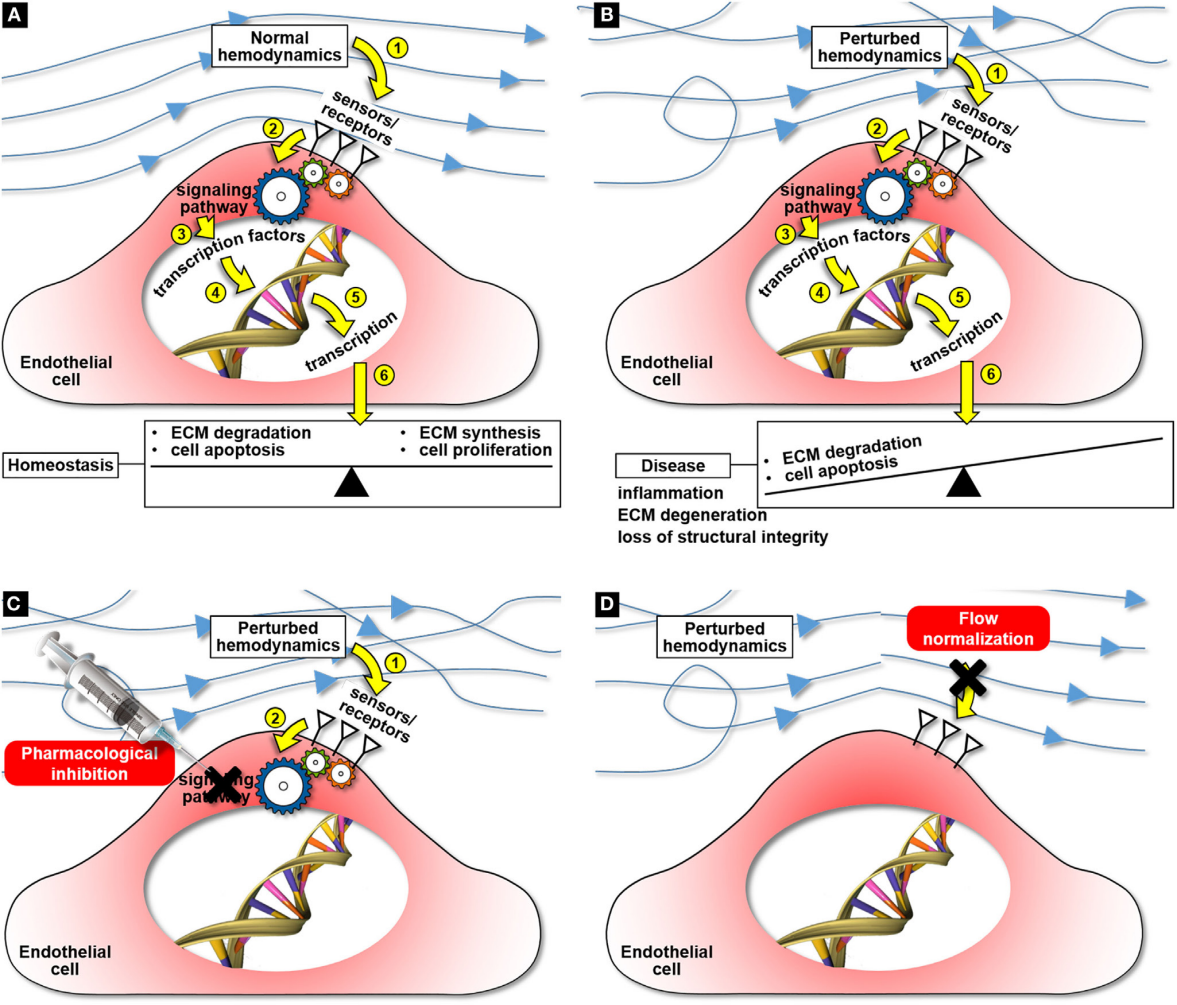
**Cardiovascular mechanobiology and potential mechanobiology-based therapies for cardiovascular disease: maintenance of tissue homeostasis under normal hemodynamics (A), hemodynamic pathway of disease development under perturbed flow conditions (B), targeted cell therapy aimed at blocking the flow-induced disease pathway (C), and flow normalization aimed at preventing the activation of the flow-induced disease pathway (D)**.

Mechanical forces are powerful regulators of biology and disease. In the vasculature, the expression of particular cellular phenotypes appears to depend not only on a combination of intrinsic genetically programed biology but also on local hemodynamic environmental factors induced by blood flow (Nerem and Girard, 1990). A major component in the spectrum of forces experienced by cardiovascular tissue is the friction force exerted by the blood flow on the endothelium. Through multiple dedicated receptors, endothelial cells are able to sense the magnitude and directionality of this force and of the resulting wall shear stress (WSS), and to transduce this mechanical signal into biochemical signals, altering in turn cellular function. Interestingly, while physiologic WSS maintains vascular homeostasis (Figure 1A), WSS abnormalities often correlate with disease states (Figure 1B). While hemodynamic pathways have been identified in some cardiovascular pathologies such as calcific aortic valve disease (CAVD) (Butcher et al., 2008), aneurysms (Humphrey et al., 2015), atherosclerosis (Cunningham and Gotlieb, 2005), and intimal hyperplasia (IH) (Haruguchi and Teraoka, 2003), the clinical management of these disorders remains focused on addressing their symptoms *via* aggressive modalities rather than blocking the flow-induced pathological cascade. As a result, current treatments are often palliative and ignore the driving biological processes. In this context, the elucidation of the cause-and-effect relationships between cardiovascular biology and hemodynamics has the potential to advance the understanding of disease progression and to enable new diagnosis and treatments.

## Mechanobiology: A Platform for Effective Disease Management Strategies

Knowledge gained from mechanobiology could contribute to the development of more effective treatment modalities articulated around two basic strategies.

### Identification of Target Molecular Pathways for Non-Invasive Disease Management

#### Principle

The effectiveness of a pharmacological treatment depends on the ability to identify potential target molecules involved in the early stage of the disease before the pathology attains a point of no return. Unfortunately, the current state of the science on many cardiovascular disorders still does not permit to support a particular pharmacological target. As it is the case with many cardiovascular pathologies, the sensing of flow abnormalities and their transduction into downstream pathological cascades are the initial triggering events of disease development. The identification of key flow-sensitive molecules responsible for transducing alterations in the surrounding mechanical environment into the initial inflammation and remodeling biological responses would enable the development of targeted, non-invasive pharmacological cellular therapies aimed at blocking the mechanobiological cascade (Figure 1C).

#### Example: Calcific Aortic Valve Disease

While the formation of calcific nodules on the aortic surface of the valve leaflets has been described historically as an age-related degenerative disorder, recent progress in valvular mechanobiology has shed new light on the potential role played by blood flow abnormalities in the early inflammatory precursor events to calcification (Balachandran et al., 2011). The identification of TGF-β1 and BMP-4 as key flow-sensitive molecules and key drivers of valvular inflammation (Sucosky et al., 2009) and the ability of their inhibitors (SB-431542 and noggin, respectively) to reduce or completely block the downstream flow-induced inflammatory response (Hoehn et al., 2010; Sun and Sucosky, 2015) have suggested the use of such inhibitors in targeted pharmacological modalities aimed at preventing the onset or slowing the progression of CAVD.

### Hemodynamic Normalization

#### Principle

Another potential strategy is to address directly the flow abnormalities responsible for the downstream biological cascade and to reverse the local hemodynamics to its physiologic baseline level. The identification of the specific hemodynamic factors (e.g., WSS magnitude/directionality, pressure) responsible for the pathological response and their normalization could potentially slow down the disease process and limit or completely block its development (Figure 1D).

#### Example: Intimal Hyperplasia in Vascular Access

The main mode of failure of hemodialysis vascular access is the progressive occlusion of the vein through which arterial flow is shunted. The initial event leading to stenosis is IH, a disorder causing the thickening of the innermost layer of the vein wall and whose etiology has been associated with the flow disturbances caused by the redirection of the arterial flow through the vein (Glagov, 1994). Strategies aimed at preserving the shunt while limiting the remodeling response of the vein *via* flow normalization have been recently developed and are currently being tested. One such effort consists of implanting a valve device at the anastomosis in order to isolate the arteriovenous shunt from the rest of the circulation (thus normalizing venous flow) between hemodialysis sessions and to allow the passage of blood through the graft (thus achieving normal vascular access) during hemodialysis sessions (McNally et al., 2014). Rather than blocking the remodeling pathway leading to stenosis pharmacologically, this strategy relies on the normalization of the flow to block the downstream biological cascade.

## Challenges, Knowledge Gap, and Needs

The success of the strategies outlined above relies on the ability to resolve the mechanobiological processes involved in disease and to translate the knowledge gained into a clinical solution. These points are discussed below and summarized in Table 1.

**Table 1:** **Clinical translation of mechanobiology: research needs, enabling technologies and methodologies, and challenges**.

Research needs	Hemodynamic characterization	Mechanobiological response elucidation	Clinical translation
Enabling methodologies and technologies	Computational modeling (CFD, FSI) *In vitro* measurements (LDV, PIV) *In vivo* measurements (4D MRI)	Bioreactor technology (shear stress bioreactors, organ culture systems, microfluidic devices) Tissue/cell cultures Biological assessment (RT-PCR, immunoblotting, immunostaining, zymography)	Biostatistics Molecular inhibitors identification (*in vitro* and *in vivo* studies) Flow normalization device design and development
Challenges	Patient-specific anatomies and boundary conditions Spatial and temporal resolutions Cost	Maintenance of sterility Bioreactor level of sophistication Multi-scale biological characterization (cell/tissue-level)	Drug safety Device thrombogenicity, biocompatibility FDA approval Cost Effectiveness

*CFD, computational fluid dynamics; FSI, fluid–structure interaction; LDV, laser Doppler velocimetry; PIV, particle image velocimetry; MRI, magnetic resonance imaging; RT-PCR, real-time polymerase chain reaction*.

### Hemodynamic Characterization

The first requirement in mechanobiological studies is the definition of the normal (i.e., baseline) hemodynamic stress state and of the hemodynamic alterations triggering a pathological state. The characterization of blood flow is challenging due to its three-dimensionality, unsteadiness, turbulence, and strong coupling with the surrounding compliant vasculature (Dasi et al., 2009). Nevertheless, the emergence of state-of-the-art imaging techniques and flow measurement and modeling tools has provided new opportunities to carry out this characterization. In the clinic, magnetic resonance imaging has been used to visualize and quantify blood flow in individual patients (Markl et al., 2016). While this approach is effective in capturing global patient-specific flow characteristics, most equipment provides relatively low spatial resolution, which restricts its implementation to large anatomies and prevents the precise quantification of velocity gradients and hemodynamic stresses. In the laboratory, standard flow measurements techniques, such as laser Doppler velocimetry and particle image velocimetry, can be used to characterize the flow at higher temporal and spatial resolutions (Sengupta et al., 2012; Seaman et al., 2014, 2015). However, such techniques often require complex setups and flow loops aimed at mimicking the native flow and anatomy and only provide flow information at discrete points or discrete sections within the anatomy. Lastly, computational fluid–structure interaction models have been developed to capture blood flow at high resolutions in compliant anatomies (Peskin and McQueen, 1992; Borazjani et al., 2013; Cao and Sucosky, 2015, 2016; Cao et al., 2015). However, the current computational capabilities still prevent the integration of realistic anisotropic and non-linear material models, complex patient-specific anatomies, and flow boundary conditions. The continuous increase in computational power and the increasing number of commercial fluid–structure interaction solvers are expected to lift most of those limitations in the near future.

### Tissue and Cell Exposure to Mechanical Forces

The characterization of the biological response following tissue or cell exposure to a particular mechanical signal is another important component of mechanobiology. Typically, cells and tissue specimens have been conditioned mechanically using commercial and in-house bioreactors. While early devices (e.g., parallel flow chamber, pressure chamber) were able to generate only basic mechanical stimuli (e.g., unidirectional steady WSS, steady pressure), current bioreactors are able to mimic more closely the complexity of the native hemodynamic environment. Examples of such devices are single and double cone-and-plate bioreactors to expose vascular tissue to single-sided or double-sided pulsatile WSS (Sucosky et al., 2008; Sun et al., 2011), stretch bioreactors to subject cells or tissue to desired cyclic stretch waveforms (Balachandran et al., 2006), and stretch-and-pressure bioreactors to selectively apply either cyclic pressure, cyclic stretch, or both on tissue samples (Thayer et al., 2011). Following mechanical conditioning, the biological characterization is typically carried out using standard biological assays and protein quantification methods (e.g., immunoblotting, immunohistochemistry, zymography, RT-PCR). The implementation of such devices has shed new lights on the mechano-etiology of CAVD (Balachandran et al., 2009, 2010; Sun et al., 2012, 2013), aortic dilation (Atkins and Sucosky, 2014; Atkins et al., 2014, 2016), and atherosclerosis (Dai et al., 2004).

### Clinical Translation

While mechanobiological studies have already contributed immensely to the understanding of cardiovascular pathologies, only few have been translated into clinical solutions. Major challenges include the complexity of interpretation of biological data, the assessment of the isolated and synergistic roles of mechanosensitive molecules in the disease process, the identification of effective, but safe, molecular inhibitors aimed at blocking the mechanobiological cascade, and the design of effective procedures and devices to normalize blood flow. While those complex issues are still current, the recent realization of the potential use of mechanobiology as a discovery tool for novel treatments and diagnosis modalities has motivated some collaborative efforts between the clinical and engineering fields. Those synergies are necessary to complement the basic science of mechanobiology and to elevate it to effective clinical solutions.

## Conclusion

With the continuous progress in flow characterization techniques, bioreactor technologies and biological assessment methodologies, mechanobiology has emerged as a potential tool to deliver the next generation of therapies in cardiovascular disease. The characterization of mechanical cues promoting cardiovascular pathogenesis, the identification and modeling of key mechanosensitive molecules and molecular pathways involved in the early stage of disease, and the integration of this knowledge into patient-specific flow models could provide new therapeutic modalities and predictive capabilities that will transform clinical decision-making and personalized care in cardiovascular medicine.

## Author Contributions

SA and AM wrote the paper and share first authorship. PS wrote the paper and conceived the work.

## Conflict of Interest Statement

The authors declare that the research was conducted in the absence of any commercial or financial relationships that could be construed as a potential conflict of interest.
